# Value of multi-detector computed tomography angiography in predicting acute cardiac events in patients with type 2 diabetes

**DOI:** 10.3892/etm.2014.1502

**Published:** 2014-01-27

**Authors:** DALIANG LIU, HUIJUAN JIA, WEI LIU, DAQING MA, GUANGSHAN TAN, WEN HE, YUCUN FU, LE-XIN WANG

**Affiliations:** 1 Department of Radiology, Beijing Friendship Hospital, Capital Medical University, Beijing 100050, P.R. China; 2 Department of Radiology, Liaocheng People’s Hospital and Liaocheng Clinical School of Taishan Medical University, Liaocheng, Shandong 252000, P.R. China; 3 Central Experimental Laboratory, Liaocheng People’s Hospital and Liaocheng Clinical School of Taishan Medical University, Liaocheng, Shandong 252000, P.R. China; 4 School of Biomedical Sciences, Charles Sturt University, Wagga Wagga 2650, Australia

**Keywords:** diabetes mellitus, multi-detector computed tomography angiography, acute coronary syndrome, atherosclerotic plaque

## Abstract

The aim of the present study was to investigate the predictive value of multi-detector computed tomography angiography (MDCTA) on acute coronary artery events in patients with type 2 diabetes mellitus (T2DM). MDCTA was performed in 150 patients with T2DM (males, 74; mean age, 66±6.7 years for all patients) that had experienced atypical chest pains. After a follow-up period of at least 2 years, 55 patients were excluded from the study as they did not exhibit any coronary events. The remaining 95 patients were divided into the study group (n=28), that had experienced an acute coronary event such as acute coronary syndrome, or the control group (n=67) that had stable angina. There were no statistically significant differences in the degree of coronary artery lumen stenosis between the study and control groups (P=0.380). The proportion of calcified plaques in the study group was significantly lower compared with the control group (13.6 vs. 53.2%; P<0.001), while the proportion of soft plaques in the study group was significantly higher compared with the control group (37 vs. 9.3%; P<0.001). Type III plaques showed a sensitivity of 76.2% and a negative predictive value of 64.5% for acute coronary events. By contrast, type IV plaques had a sensitivity of 52.6% and a positive predictive value of 63% for chronic coronary events. Therefore, the results of the present study indicate that MDCTA may be used as a noninvasive modality for evaluating and predicting vulnerable coronary atherosclerosis plaques in patients with T2DM.

## Introduction

Type 2 diabetes mellitus (T2DM) is a major risk factor for cardiovascular disease and is associated with significant cardiovascular morbidity and mortality (1). Patients with T2DM have an increased risk of acute coronary events (1). Acute coronary events are often caused by the rupture of unstable coronary atherosclerotic plaques and coronary stenosis (2,3). Therefore, assessment of atherosclerotic plaque morphology and pathological features has become an important part of the clinical investigation of coronary artery disease (4). Multi-detector computed tomography angiography (MDCTA) has been increasingly used in the evaluation of coronary arteries (5). In an acute setting, MDCTA is associated with 95% sensitivity and 90% specificity in diagnosing non-ST-elevation myocardial infarction and unstable angina pectoris (6). The ability to detect not only coronary stenosis, but also non-obstructive coronary atherosclerotic plaques using a non-invasive method indicates that MDCTA imaging is a potentially valuable tool for risk stratification. In previous studies, MDCTA has been used to predict cardiac events and prognosis in patients with suspected coronary artery disease (7–14). However, there is limited information on the predictive value of MDCTA on acute coronary events in patients with T2DM.

Therefore, the purpose of the present study was to investigate the MDCTA characteristics of coronary plaques in patients with T2DM. The sensitivity and specificity of MDCTA in predicting acute coronary events in these patients was also evaluated.

## Subjects and methods

### Study population

**Figure 1:**
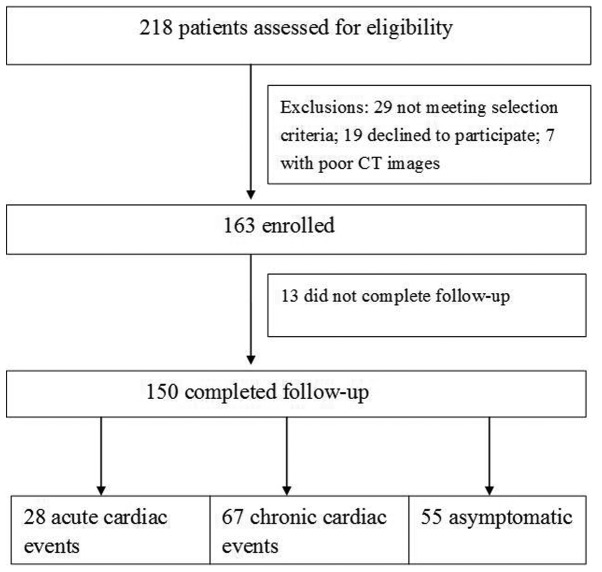
Flow chart of the study participants.

The study was approved by the Institutional Ethics Committee of Liaocheng People’s Hospital (Liaocheng, China) and written informed consent was provided by all the participants. Between February 2007 and November 2009, 218 consecutive patients with T2DM were referred to our department at Liaocheng People’s Hospital (Liaocheng, China) for coronary MDCTA due to nonspecific chest pain, exertional dyspnea or ST-T depression or flattening observed on an electrocardiogram (ECG). These patients were screened for the present study. Patients who had undergone previous coronary balloon angioplasty, stenting or coronary artery bypass grafting were excluded. Additional exclusion criteria were as follows: i) Heart rate >90 beats/min, atrial fibrillation or other arrhythmias; ii) renal dysfunction (serum creatinine ≥120 mmol/l); iii) other chronic illnesses, including severe respiratory insufficiency and hyperthyroidism; and iv) patients who were unable to provide written consent. In total, 150 patients were recruited and 58 patients were excluded in the study. The reasons for exclusion are listed in Fig. 1.

### MDCTA protocol

The first 95 patients were scanned by a Philips Brilliance 64-detector CT (Philips Medical Systems, Eindhoven, Netherlands). Prior to the scans, β-blockers were administered to patients whose heart rate was ≥70 beats/min, and 0.3 mg nitroglycerin was sublingually administered to all patients 15 min prior to the scans. Retrospective ECG-gated helical MDCTA was performed with a 64-detector CT. The scan parameters were 64×0.625 mm collimation, 120 kV tube voltage, 400–600 mAs tube current, 0.42 sec rotation time and 0.2 pitch. Data acquisition was completed within 4.1–5.9 sec with a radiation dose of 13.9–16.8 mSv (median, 15.1 mSv). In the remaining 55 patients, a prospective ECG-gated scan was performed with a 128-detector CT and the heart rate was restricted to within 60–70 beats/min. The scan parameters were 120 kV tube voltage, 200 mAs tube current, 128×0.625 mm collimation, 0.18–0.27 sec rotation time and 0.2 pitch. Scanning was completed within 3.9–6.8 sec with a radiation dose between 3.16 and 4.14 mSv (median, 3.6 mSv).

A 50–60 ml (dependent on body mass index) bolus of iodinated contrast agent (iopamidol; 370 mg iodine/ml; Bracco Sine Pharmaceutical Corp. Ltd, Pudong, China) was injected into the antecubital vein at a flow rate of 4–5 ml/sec. The scanning range was between the tracheal bifurcation and 10 mm below the inferior cardiac apex. Best quality images were selected for evaluation and other phases or ECG editing was performed if required. All initial data sets were transferred to a post-processing workstation (Brilliance-workshop; Philips Medical Systems) for image analysis. Alternative image reconstruction methods for evaluation of coronary arteries and plaques included maximum intensity projection, multi-planar reconstruction, curvature plane reconstruction and volume rendering.

### Stenosis and plaque analysis

Two cardiovascular radiologists analyzed the images independently. The radiologists were blinded to the medical histories, clinical diagnoses and results of other investigations for all the patients. In cases of disagreement, the features of plaque and stenosis evaluations were re-evaluated for a consensus judgment.

**Figure 2:**
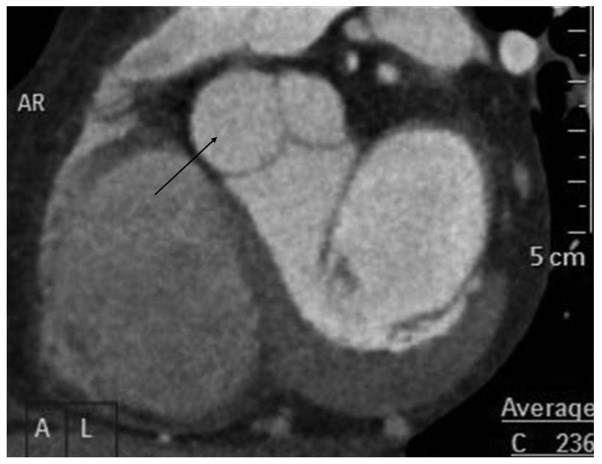
A 51-year-old female with an 8-year history of T2DM. Soft plaques were identified in the left descending coronary artery with an eccentric and unsmooth surface (black arrow). The CT value was 28 Hu. CT, computed tomography; T2DM, type 2 diabetes mellitus.

**Figure 3:**
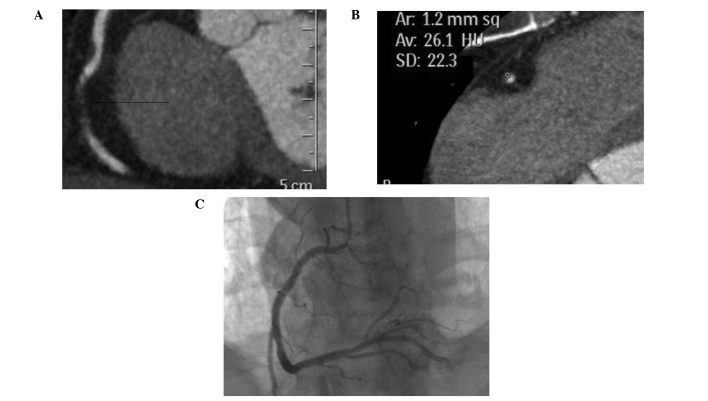
A 57-year-old female with an 11-year history of T2DM. (A and B) Soft plaques in the middle segment of the right coronary artery with an eccentric and smooth surface (black arrow). The CT value was 26 Hu. (C) The plaque was identified through routine coronary angiography. CT, computed tomography; T2DM, type 2 diabetes mellitus.

Subsequently, the type of plaque was determined to be non-calcified, calcified or mixed (15). Non-calcified plaques had a lower density compared with the contrast-enhanced lumen, while calcified plaques had a higher density. Mixed plaques exhibited soft and calcified elements within a single plaque. Measurements were performed in axial and multiplanar reconstruction images and four points were selected randomly. Regions of interest of >1.0 mm^2^ (at least 3 contiguous pixels; area, 1.03 mm^2^) and the smallest CT value was defined as the size of the plaques (Figs. 2 and 3). The coronary artery plaques were classified into four types as previously described (16,17): Type I concentric lesions, type II eccentric lesions with a wide base but smooth margin, type III eccentric lesions with a narrow base and rough surface and type IV long segments of irregular lesions.

The number of affected coronary vessels, segments and plaques, as well as the types of plaques and grading of stenosis caused by the plaques were evaluated. Coronary arteries were divided into a 15 segment model of the American Heart Association (18). The involved vessels were classified as single, double or triple vessels. The degree of stenosis was defined as the percentage of stenosis and adjacent normal vessel lumen (normal or mild, <50% stenosis; moderate, 50–75% stenosis; severe, ≥75% stenosis) (19).

### Follow-up

Follow-ups were conducted by structured telephone interviews with the patients or relatives that understood the patients’ condition. The follow-up questionnaires included the general health of the patients, use of medications, cardiovascular events, including hospital admissions, coronary artery angiography or stenting, or coronary artery bypass grafting. The interviews were conducted every 6 months for at least 24 months.

Acute coronary events were defined as acute coronary syndrome (ST- or non-ST elevation myocardial infarction or unstable angina) or sudden cardiac mortality (study group). Patients with stable angina or with angiographically identified coronary artery stenosis requiring elective coronary stenting or bypass grafting 6 months following MDCTA, were considered as having chronic coronary events (control group) (14).

### Statistic analysis

Data are expressed as the mean ± SD. χ^2^-test was used to compare the categorical data between the study and control groups. P<0.05 was considered to indicate a statistically significant difference. All statistical analyses were performed using the SPSS statistical package (version 11.5 for Windows; SPSS, Inc., Chicago, IL, USA).

## Results

### General observations

A total of 163 patients were recruited to the study, but only 150 patients completed the follow-up (mean follow-up time, 30±5.6 months; range, 24–57 months; Fig. 1). The baseline characteristics of these patients are summarized in Table I.

During the follow-up period, acute coronary events occurred in 28 patients (18.7%), including acute myocardial infarction in eight individuals (5.3%), unstable angina in 19 patients (12.7%) and sudden mortality in one patient (0.7%). Within the first six months of the follow-up period, 15 patients exhibiting acute coronary events underwent coronary stenting and eight received coronary bypass grafting. Chronic coronary events occurred in 67 patients (44.7%), including stable angina pectoris in 58 individuals, elective coronary stenting in seven patients and elective coronary bypass grafting in two patients. The remaining 55 patients (36.7%) were asymptomatic and free of coronary events during the follow-up period.

### Coronary artery plaques on MDCTA images

A total of 420 segments were analyzed in the study group and 1,005 segments were analyzed in the control group (Table II). Triple-vessel diseases were identified in 67.8% of the study group and 68.6% of the control group (P=0.380). A moderate to severe degree of coronary stenosis was identified in 89.8% of the study group and 88% of the control group (P=0.380).

The numbers and characteristics of the coronary plaques in the study and control groups are shown in Table III. In the study group, there were 236 non-calcified, 315 mixed and 87 calcified plaques. By contrast, in the control group, the numbers of non-calcified, mixed and calcified plaques were 148, 596 and 843, respectively. The proportion of calcified plaques in the study group was significantly lower compared with the control group (13.6 vs. 53.2%; P<0.01). In addition, the proportion of non-calcified plaques in the study group was significantly higher compared with the control group (37 vs. 9.3%; P<0.001).

### Plaque morphology and acute coronary events

The numbers of various plaque types observed following MDCTA are listed in Table IV. In the study group, there was no statistically significant difference in plaque type between the patients with moderate or severe coronary stenosis (P=0.349). However, in the control group, there were statistically significant differences in plaque type among the patients with various grades of stenosis (P<0.001).

As shown in Table IV, there was no statistically significant difference in the proportion of type I plaques between the study and control groups (P>0.05). However, the proportion of type III plaques in the study group was higher compared with the control group, whereas the proportion of type II and IV plaques was lower (P<0.01).

Using type III plaques to predict acute coronary events, the sensitivity and specificity levels were 63.8 and 76.2%, respectively. However, using type II and IV plaques to predict chronic coronary events, the sensitivity and specificity levels were 91.5 and 79%, respectively.

## Discussion

The main observations of the present study were as follows. Firstly, the proportion of non-calcified and type III plaques in patients with acute coronary events was higher compared with patients with stable angina. Secondly, the proportion of calcified plaques in patients with stable angina was higher compared with patients with acute coronary events. Finally, the sensitivity and specificity levels of type III plaques in predicting acute coronary events were 63.8 and 76.2%, respectively, whereas the sensitivity and specificity levels of type II and IV plaques in predicting chronic coronary events were 91.5 and 79%, respectively. These observations indicate that non-invasive MDCTA may be used to evaluate the vulnerability of coronary plaques in patients with T2DM and as a tool to predict acute coronary events.

Previous studies have found that numerous coronary lesions in patients with coronary heart disease are non-obstructive and vessels with mild to moderate stenosis are responsible for cardiac events (20,21). The vulnerability of intracoronary lesions is a key factor for acute cardiac events in these patients with mild to moderate stenosis (20,21). Acute coronary syndrome is often caused by rupture of coronary artery atherosclerosis plaques, rather than lumen stenosis (22). Therefore, early detection of vulnerable or unsteady plaques is important in guiding prevention and treatment of acute cardiac events. Non-calcified plaques are unsteady and have been commonly observed in patients with acute coronary syndrome (23,24). Consistent with previous studies, the results of the present study demonstrate that there is little difference in stenosis severity between patients with acute and chronic coronary events. By contrast, the types of coronary plaques detected by MDCTA appear to be associated with coronary events. The current study also identified that patients with acute coronary syndrome exhibited an increased number of non-calcified plaques and fewer calcified plaques compared with patients with stable angina. These results indicate that in patients with T2DM, analysis of stenosis severity using MDCTA alone may be insufficient. Evaluation of plaque morphology and vulnerability using MDCTA offers additional information on the future risk of acute coronary events.

The reliability of MDCTA in assessing coronary plaque stability has been increasingly studied in recent years. A previous study by Motoyama *et al* (25) demonstrated that MDCTA can be used to accurately assess plaque composition by measuring the CT value of the plaque on thin-CT images. Falk *et al* (26) identified that eccentric lesions were an index of unsteady plaques. The lipid core of the plaque is present in the inner lining of the coronary lumen and is prone to rupture under the impact of blood flow. Ruptured plaques adsorb platelets, resulting in new clot formation, obstruction of coronary flow and acute coronary syndrome or sudden mortality (27). In the present study, the coronary plaques were divided into four types as previously described (16,17). Type III plaques, those with an eccentric center and rough surface, appear to be associated with acute coronary events. The predictive sensitivity and specificity levels of type III plaques for acute coronary events were 63.8 and 76.2%, respectively. By contrast, eccentric lesions with a wide base but smooth margin (type II plaques) and long segments of irregular lesions (type IV plaques) are relatively stable plaques and demonstrated 91.5% sensitivity and 79% specificity in predicting chronic coronary events.

A potential limitation of the present study regarding the first 95 patients is that at the beginning of the study, retrospective ECG-gated helical MDCTA was performed using a 64-detector CT. In the remaining 55 patients, prospective ECG-gated scans were performed using a 128-detector CT. The imaging quality of the 128-detector MDCTA is generally superior to the 64-detector MDCTA and the dose of radiation is also lower (5,27). In total, 7 patients were excluded from the study due to poor imaging quality and these patients were scanned with the 64-detector MDCTA.

In conclusion, MDCTA is a non-invasive tool that can be used to measure the severity of coronary stenosis and assess the morphology of coronary plaques. In patients with T2DM, eccentric plaques with rough surfaces have a moderate sensitivity and specificity in predicting acute coronary events. Eccentric plaques with smooth surfaces and long segments of irregular coronary lesions are more likely to be associated with chronic coronary events. These observations indicate that morphology analysis using MDCTA may improve coronary risk stratification in patients with T2DM.

## Figures and Tables

**Table I:** Baseline characteristics.

Characteristics	Study group (n=28)	Control group (n=67)	P-value
Age, years	64.7±14.6	63.6±15.3	NS
Gender, male, n (%)	16 (57.1)	29 (43.3)	NS
BMI, kg/m^2^	25.8±3.54	25.3±1.49	NS
Other cardiovascular risk factors, n (%)			
Hypertension	17 (60.7)	42 (62.7)	NS
Hypercholesterolemia	13 (46.4)	32 (47.8)	NS
Smoking	15 (53.6)	34 (50.7)	NS
Family history of CHD	9 (32.1)	21 (31.3)	NS
Clinical presentation, n (%)			
Abnormal ECG	24 (85.7)	46 (68.7)	NS
Atypical angina	8 (28.6)	46 (68.7)	NS
Nonspecific chest pain	17 (60.7)	38 (56.7)	NS
Dyspnea	18 (64.3)	45 (67.2)	NS
Diabetes treatment, n (%)			
Oral agents	2 (7.1)	12 (17.9)	NS
Oral agents plus insulin	26 (92.8)	55 (82.8)	NS

BMI, body mass index; CHD, coronary heart disease; NS, no significant difference; ECG, electrocardiogram.

**Table II:** Comparison of coronary lesions and degree of coronary stenosis between groups.

Coronary lesions	Study group (n=28)	Control group (n=67)	P-value
Vessels involved, n (%)			
Single	4 (14.4)	8 (12)	NS
Double	5 (17.8)	13 (19.4)	NS
Triple	19 (67.8)	46 (68.6)	NS
Stenosis grade, n (%)			
<50%	43 (10.2)	121 (12.0)	NS
50–75%	263 (62.6)	641 (63.8)	NS
≥75%	114 (27.2)	243 (24.2)	NS

NS, no significant difference.

**Table III:** Comparison of plaque numbers and characteristics between the acute and chronic groups.

Plaque characteristics	Study group (plaques, 638)	Control group (plaques, 1,586)	P-value
Non-calcified, n (%)	236 (37)	148 (9.3)	<0.001
Mixed, n (%)	315 (49.4)	595 (37.5)	<0.001
Calcified, n (%)	87 (13.6)	843 (53.2)	<0.001

**Table IV:** Comparison of plaque morphology between the study and control groups.

Plaque morphology	Study group (plaques, 638)	Control group (plaques, 1,586)	P-value
Type I, n (%)	38 (6)	89 (5.6)	0.825
Type II, n (%)	65 (10.2)	637 (42.2)	<0.001
Type III, n (%)	486 (76.2)	276 (17.4)	<0.001
Type IV, n (%)	49 (7.6)	584 (36.8)	<0.001
